# Contrast-Enhanced Mammography in Breast Lesion Assessment: Accuracy and Surgical Impact

**DOI:** 10.3390/tomography11080093

**Published:** 2025-08-20

**Authors:** Graziella Di Grezia, Sara Mercogliano, Luca Marinelli, Antonio Nazzaro, Alessandro Galiano, Elisa Cisternino, Gianluca Gatta, Vincenzo Cuccurullo, Mariano Scaglione

**Affiliations:** 1 Department of Life Sciences, Health and Healthcare Professions Link Campus University, 00165 Rome, Italy; 2 Breast Unit, Radiology and Diagnostic Imaging Department, AORN Santi Anna e Sebastiano, 81100 Caserta, Italy; 3 Diagnostic Imaging, University of Campania Luigi Vanvitelli, 80138 Naples, Italy; 4 Independent Researcher, 83100 Avellino, Italy; 5 Department of Radiology, P.O. A. Perrino Hospital, 72100 Brindisi, Italy; 6 Department of Advanced Medical and Surgical Sciences, University of Campania Luigi Vanvitelli, 80138 Naples, Italy; 7 Department of Precision Medicine, University of Campania Luigi Vanvitelli, 80138 Naples, Italy; 8 Department of Radiology, University of Sassari, 07100 Sassari, Italy

**Keywords:** breast cancer, contrast enhanced mammography, breast imaging, ultrasound, mammography

## Abstract

Background: Accurate preoperative tumor sizing is critical for optimal surgical planning in breast cancer. Contrast-enhanced mammography (CEM) has emerged as a promising modality, yet its accuracy relative to conventional imaging and pathology requires further validation. Objective: To prospectively evaluate the dimensional accuracy and reproducibility of CEM compared to mammography and ultrasound, using surgical pathology as the reference standard. Methods: A total of 205 patients with 267 breast lesions underwent preoperative CEM, mammography, and ultrasound. Tumor sizes were measured independently by two radiologists. Accuracy was assessed via mean absolute error (MAE), Pearson and Spearman correlations, and inter-reader agreement evaluated by intraclass correlation coefficient (ICC) and Gwet’s AC1. Sensitivity analyses included bootstrap confidence intervals and log-transformed data. The surgical impact of additional lesions detected by CEM was also analyzed. Results: CEM showed superior accuracy with a mean absolute error of 0.46 mm (95% CI: 0.24–0.68) compared to mammography (4.06 mm) and ultrasound (3.52 mm) (*p* < 0.00001). Pearson’s correlation between CEM and pathology was exceptionally high (r = 0.995; 95% CI: 0.994–0.996), with similar robustness after log transformation. Inter-reader agreement for CEM was excellent (ICC 0.93; Gwet’s AC1 ~0.96, 95% CI: 0.93–0.98). CEM detected additional lesions in 13.1% of patients, leading to altered surgical management in 6.4%. Background parenchymal enhancement was independently associated with measurement error. Conclusions: CEM provides highly accurate and reproducible tumor size estimation superior to conventional imaging modalities, with potential clinical impact through detection of additional lesions. Its ability to detect additional lesions not seen on mammography or ultrasound has direct implications for surgical decision making, with the potential to reduce reoperations and improve oncologic and cosmetic outcomes. However, high correlation values and selective patient cohorts warrant cautious interpretation. Further multicenter studies are needed to confirm these findings and define CEM’s role in clinical practice.

## 1. Introduction

Breast cancer remains the most frequently diagnosed malignancy and a leading cause of cancer-related mortality among women worldwide, with early and accurate assessment playing a pivotal role in clinical decision making and prognosis [1]. Preoperative estimation of tumor size is crucial for surgical planning, particularly in breast-conserving surgery (BCS), where achieving clear margins while preserving cosmetic outcomes is essential [2]. Discrepancies between radiologic and histopathologic measurements may result in incomplete tumor excision, reoperations, or overtreatment.

Mammography (MG) and ultrasound (US) are widely used for initial lesion detection and characterization; however, both modalities have limitations in accurately estimating tumor size. Mammography may underestimate lesion extent due to tissue overlap and lack of functional information, especially in dense breasts [3,4]. Ultrasound, while accessible and operator-friendly, is often limited by operator dependency and challenges in delineating non-mass lesions or multifocal disease [5].

Contrast-enhanced mammography (CEM) is an increasingly adopted technique combining conventional mammographic imaging with intravenous iodinated contrast administration to highlight areas of increased vascularity typically associated with malignancy. By acquiring low-energy and recombined images, CEM provides both morphological and functional information, enhancing lesion conspicuity and potentially improving diagnostic accuracy [6]. Several studies have demonstrated that CEM exhibits superior sensitivity compared to conventional mammography and comparable performance to breast magnetic resonance imaging (MRI), particularly in detecting multifocal and multicentric disease [7,8,9]. Moreover, CEM offers logistical advantages over MRI, including shorter acquisition times, lower costs, and greater accessibility [10].

Despite growing clinical adoption, robust prospective data evaluating CEM’s dimensional accuracy for tumor size estimation compared with standard imaging modalities and surgical pathology remain limited [11,12,13,14,15,16,17,18]. Additionally, biological and technical variables such as tumor subtype, breast density, and background parenchymal enhancement (BPE) may influence imaging accuracy and warrant further detailed investigation [19,20,21,22,23,24,25,26]. While several prior studies have assessed the diagnostic performance of CEM compared to FFDM, ultrasound, and MRI, relatively few have focused specifically on the dimensional accuracy of CEM using surgical pathology as a rigorous reference standard. However, existing studies often lack direct head-to-head comparisons with both FFDM and US in the same patient cohort, and frequently include small or heterogenous samples. The present study addresses these gaps by systematically evaluating the size accuracy of CEM, FFDM, and US in 205 consecutive patients (267 lesions), all with surgical pathology confirmation. By including a robust set of clinical and molecular subgroups, as well as inter-reader agreement metrics and error stratification, we offer new insights into the practical reliability of CEM in preoperative staging. This approach positions CEM as a potential intermediate solution between conventional imaging and MRI for surgical planning. Furthermore, we investigate the influence of clinical and biological variables on measurement accuracy and assess the impact of CEM-detected additional lesions on surgical decision making.

## 2. Materials and Methods

### 2.1. Study Design and Setting

This prospective, single-center observational study was conducted at the Interventional Senology Unit of the ‘A. Perrino’ Hospital, Brindisi, Italy. The study protocol was approved by the local Institutional Review Board and local ethical committee, and all patients provided informed consent before participation. The study adhered to the Declaration of Helsinki and complied with STARD 2015 guidelines for diagnostic accuracy studies [27].

### 2.2. Patient Selection

Between May 2022 and June 2023, consecutive female patients with histologically confirmed breast cancer scheduled for surgery were enrolled. Inclusion criteria were as follows:

- Availability of preoperative Contrast-Enhanced Mammography (CEM).
- Patients had undergone both full-field digital mammography (FFDM) and targeted ultrasound (US) imaging, in addition to CEM.
- Complete clinical, imaging, and histopathological data.
- Exclusion criteria included the following:
- Prior neoadjuvant chemotherapy (to avoid confounding changes in tumor size due to treatment).
- Incomplete imaging or pathological datasets.
- Suboptimal image quality, defined as inadequate lesion visibility due to motion artifacts, poor contrast uptake, incorrect positioning, or underexposure. Image quality was assessed independently by two experienced radiologists using predefined technical adequacy criteria before measurements were included in the analysis.

Patients receiving neoadjuvant chemotherapy were excluded because post-treatment pathological measurements may significantly underestimate the original tumor size, compromising the validity of imaging-pathology dimensional concordance. For this reason, only patients undergoing upfront surgery were included to ensure accurate comparison.

Of the 314 patients initially considered undergoing CEM, 109 were excluded due to these criteria (52 patients due to neoadjuvant treatment, 39 for incomplete datasets, and 18 (5.7%) due to poor image quality, as determined by consensus review). The final cohort consisted of 205 patients with 267 lesions.

### 2.3. Contrast-Enhanced Mammography System and Protocol

CEM examinations were performed using the Senographe Pristina system (General Electric Healthcare, Chicago, IL, USA) equipped with the Seno Bright software, version HD (Chicago, Illinois, USA)**,** following manufacturer recommendations.

A dual-energy technique was applied, acquiring low-energy (26–31 keV) and high-energy (45–49 keV) images of both breasts in standard craniocaudal (CC) and mediolateral oblique (MLO) views approximately two minutes after contrast injection.

An intravenous injection of non-ionic iodinated contrast agent (Iohexol 350 mg I/mL, Omnipaque, GE Healthcare) was administered at 1.5 mL/kg body weight with a 3 mL/s infusion rate, followed immediately by a 30 mL saline flush. Venous access was established with a 20G catheter connected to a dual-syringe injector.

Images were analyzed on a high-resolution workstation (Barco NV, Kortrijk, Belgium), applying the recombination algorithm embedded in the Seno Bright software to generate subtracted images, enhancing lesion conspicuity.

### 2.4. Conventional Imaging

Mammography and ultrasound were performed following institutional standard protocols using digital equipment. Mammography was performed with low-energy images (similar to standard mammograms). Ultrasound measurements recorded the longest diameter of lesion in two orthogonal planes. All FFDM and CEM images were acquired using the same unit (Senographe Pristina, GE Healthcare) as part of the standard dual-energy acquisition protocol. Ultrasound examinations were performed using the LOGIQ E10 system (GE Healthcare) equipped with a high-frequency linear transducer (12–15 MHz).

### 2.5. Lesion Measurement and Reference Standard

Two experienced breast radiologists, each with over 10 years of experience, independently measured lesion size on CEM, mammography, and ultrasound images, blinded to each other and to pathological results. The maximum lesion diameter (in mm) was recorded for each modality.

The gold standard for tumor size was the maximum diameter measured on surgical pathology specimens by a dedicated breast pathologist, using standardized grossing and measurement techniques.

To avoid measurement bias, the pathologist was blinded to imaging findings during specimen evaluation.

For multifocal or multicentric disease, only the largest invasive lesion was included in the analysis.

### 2.6. Clinical and Biological Data

Clinical variables collected included patient age, breast density (classified per BI-RADS A–D), and background parenchymal enhancement (BPE) on CEM, categorized as minimal, mild, moderate, or marked.

Histopathologic data included tumor subtype, histologic grade, estrogen receptor (ER), progesterone receptor (PR), HER2 status, and Ki67 proliferation index.

### 2.7. Statistical Analysis

Descriptive statistics were computed for all variables. Continuous variables are presented as mean (standard deviation, SD) or median (range), depending on the distribution assessed by the Shapiro–Wilk test.

Paired comparisons between imaging modalities and the pathological gold standard were performed using the Wilcoxon signed-rank test due to non-normal data distribution. Given the three pairwise comparisons between imaging modalities, Bonferroni correction was applied to adjust for multiple testing.

The primary metric of dimensional accuracy was the Mean Absolute Error (MAE), calculated as the absolute difference between imaging and pathological tumor size. MAE and other estimates are presented along with their 95% confidence intervals (CIs), calculated assuming approximate normality or via bootstrap methods when appropriate. Bootstrap resampling was performed to assess the robustness of correlation coefficients and MAE estimates without relying solely on parametric assumptions. For each lesion, MAE was defined as |Size_imaging − Size_pathology|, expressed in millimeters. Aggregated statistics (mean, SD, 95% CI) were computed within each subgroup.

Correlation between imaging measurements and pathological size was primarily assessed using Pearson’s correlation coefficient (*r*), with 95% CIs computed via Fisher’s z-transformation. Given the exceptionally high correlation observed for CEM (*r* = 0.995, 95% CI: 0.994–0.996), log-transformed analyses of lesion size measurements were also performed to confirm the stability of the results; Pearson and Spearman correlation coefficients remained similarly high (*r* ≈ 0.97, ρ ≈ 1.00), supporting robustness.

Inter-reader agreement for lesion size measurements on CEM was evaluated by the intraclass correlation coefficient (ICC) using a two-way random-effects model for absolute agreement, yielding an ICC of 0.93 (95% CI: 0.90–0.95). For categorical BI-RADS classification, inter-reader agreement was assessed using Cohen’s kappa (κ = 0.78, 95% CI: 0.69–0.87), reflecting substantial concordance. Additionally, Gwet’s AC1 was estimated at approximately 0.96 (95% CI: 0.93–0.98), indicating even stronger reliability less affected by prevalence bias. For agreement between imaging-based and pathological classification of tumor extent (i.e., unifocal, multifocal, multicentric), Cohen’s kappa statistic was calculated.

Bland–Altman plots were generated to visualize agreement between imaging modalities and pathology. Limits of Agreement (LoA) were calculated with 95% confidence intervals following Clinical and Laboratory Standards Institute (CLSI) guidelines.

Subgroup analyses investigated the impact of clinical and biological factors—including lesion type, breast density, background parenchymal enhancement (BPE), and molecular markers (ER, PR, HER2, Ki67)—on measurement accuracy using non-parametric tests and multivariable linear regression models. No formal correction for multiple comparisons was applied to these subgroup analyses, which were considered exploratory in nature

Pathological assessments were performed independently and blinded to imaging results, with no cross-reference to CEM measurements to avoid measurement dependence

A trained biostatistician was involved throughout the study design, data analysis, and interpretation phases to ensure methodological rigor.

Statistical significance was set at *p* < 0.005 in accordance with modern recommendations aimed at reducing false-positive findings. All statistical analyses were performed using R version 4.3.0 [28]. Bootstrap confidence intervals were calculated using the ‘boot’ package, inter-rater agreement metrics with the ‘irr’ package, and correlation analyses via ‘psych’ [29,30]. Exact multinomial and binomial confidence intervals were computed using LePAC and AgreeStat360, as appropriate [31,32].

## 3. Results

During the study period, 314 patients underwent Contrast-Enhanced Mammography (CEM) for preoperative breast cancer evaluation. After excluding 109 patients due to prior neoadjuvant chemotherapy (*n* = 52), incomplete pathological or imaging data (*n* = 39), or technical image quality issues (*n* = 18), the final study cohort comprised 205 patients with 267 lesions, all with complete imaging and confirmed surgical histopathology. This exclusion rate (35%) may limit the generalizability of our findings and introduces a potential selection bias.

The mean age of patients was 56.7 (11.6) years. The predominant histologic subtype was invasive ductal carcinoma (IDC), followed by invasive lobular carcinoma (ILC), ductal carcinoma in situ (DCIS), and special subtypes. Breast density distribution according to BI-RADS was as follows: A (13.1%), B (38.2%), C (29.6%), and D (19.1%). Background parenchymal enhancement (BPE) on CEM was minimal in 55.1%, mild in 24.3%, moderate in 13.4%, and marked in 7.2% of patients. The histologic subtypes of tumors included invasive ductal carcinoma (IDC) in 61% of lesions, invasive lobular carcinoma (ILC) in 28%, ductal carcinoma in situ (DCIS) in 10%, and other special subtypes comprising the remaining 1%. Tumor size distribution ranged from 5 mm to 40 mm with a median of 15 mm (Table 1).

CEM demonstrated excellent accuracy in tumor size estimation. The mean error compared to surgical pathology was +0.16 mm (95% CI: −0.03 to +0.31 mm, bootstrap) with a standard deviation of 1.45 mm (95% CI: 1.30 to 1.58 mm, bootstrap), with a median error of 0.0 mm (95% CI: −0.11 to +0.43 mm, bootstrap) and range from −14 mm to +6 mm. The distribution of errors was non-normal (Shapiro–Wilk *p* < 0.0001). The use of bootstrap confidence intervals for all estimated parameters—including mean, median, and standard deviation—further supports the statistical reliability of our results despite the non-normal error distribution.

The mean absolute error (MAE) was 0.46 mm (bootstrap 95% CI: 0.41–0.72 mm), confirming high precision and robustness of the results. Statistical testing showed no significant systematic bias (one-sample *t*-test *p* = 0.07) (Table 2).

In 162 lesions with complete measurements from all three imaging modalities, CEM significantly outperformed both mammography and ultrasound in size estimation accuracy. The MAE for CEM was markedly lower at 0.46 mm (95% CI 0.24–0.68), compared to 4.06 mm (95% CI 3.29–4.83) for mammography and 3.52 mm (95% CI 2.83–4.21) for ultrasound. Wilcoxon signed-rank tests, corrected using the Bonferroni method for multiple comparisons, confirmed that the error for CEM was significantly smaller than both mammography and ultrasound (*p* < 0.00001 for both comparisons), while no significant difference was found between mammography and ultrasound (*p* = 0.53) (Table 3).

Pearson’s correlation coefficient for CEM versus pathology was extraordinarily high (r = 0.995, 95% CI 0.994–0.996). Sensitivity analyses using log-transformed lesion sizes produced similarly strong correlations (Pearson r ≈ 0.97, Spearman ρ ≈ 1.00 CI for ρ: 0.991–1.000, bootstrap), supporting the robustness of this finding. Ultrasound and mammography showed significantly lower but meaningful correlations (r = 0.78 and 0.68, respectively; *p* < 0.0001) (Table 4).

While the near-perfect correlation of CEM measurements is exceptional and suggests remarkable measurement reliability, it is important to interpret this result with caution. The unusually high correlation may reflect sample homogeneity and meticulous measurement protocols but could also be influenced by data processing or selection bias, which warrant further validation in independent cohorts.

CEM detected 35 additional lesions (ALs), representing 13.1% (95% CI: 9.4–17.5%) of patients, which were not identified by mammography or ultrasound. The majority of these lesions (92.5%) were classified as BI-RADS 5, with the remainder distributed as BI-RADS 3 or 4. Although histopathologic confirmation of these additional lesions was limited, their detection influenced surgical planning in a subset of patients.

Surgical management remained unchanged in 192/205 (93.6%, 95% CI: 89.3–96.6%) cases after CEM. However, in 13 patients (6.4%, 95% CI: 3.4–10.7%), the surgical approach was escalated, typically to mastectomy, based on CEM findings of additional lesions, suggesting potential clinical benefit. Given the small sample size of this subgroup, these results should be regarded as preliminary and require validation in larger studies (Table 5). The agreement between CEM and pathology for tumor extent classification was substantial, with a Cohen’s κ of 0.61 (95% CI: 0.49–0.72).

**Figure 1:**
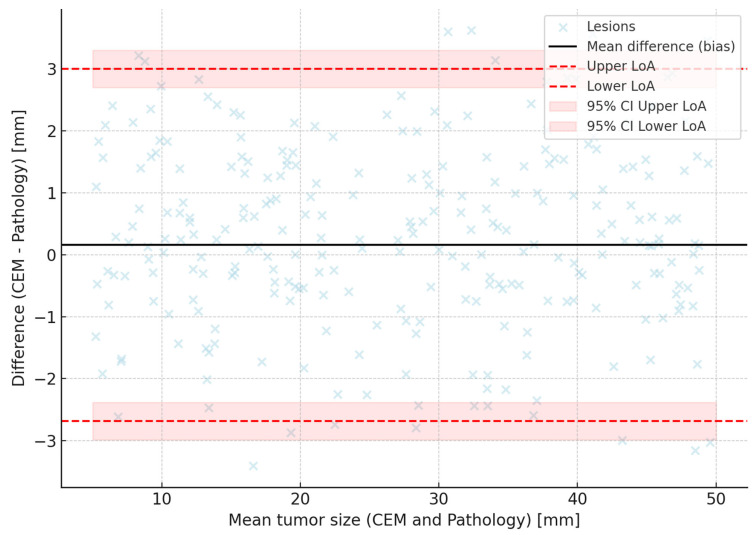
Bland–altman plot of lesion size measurements: Contrast-Enhanced Mammography (CEM) vs. pathology. This plot illustrates the agreement between tumor size measurements obtained by CEM and the gold-standard surgical pathology. The *y*-axis represents the difference between CEM and pathological sizes (CEM−Pathology) for each lesion, while the *x*-axis shows the mean tumor size of the two methods. The solid black line indicates the mean difference (bias) of +0.16 mm, demonstrating negligible systematic over- or underestimation by CEM. The dashed red lines represent the 95% limits of agreement (LoA), calculated as bias ± 1.96 times the standard deviation of the differences, ranging approximately from −2.68 mm to +3.00 mm. Shaded red bands depict the 95% confidence intervals for these limits, calculated following Clinical and Laboratory Standards Institute (CLSI) guidelines, enhancing interpretation of the precision of agreement estimates. Individual data points (light blue dots) reflect lesion-specific differences, showing a tight clustering around the bias line, indicative of high measurement consistency and precision across tumor sizes. No evident proportional bias is observed, as differences do not systematically vary with lesion size. This analysis confirms excellent concordance between CEM and pathology in preoperative tumor size estimation.

**Figure 2:**
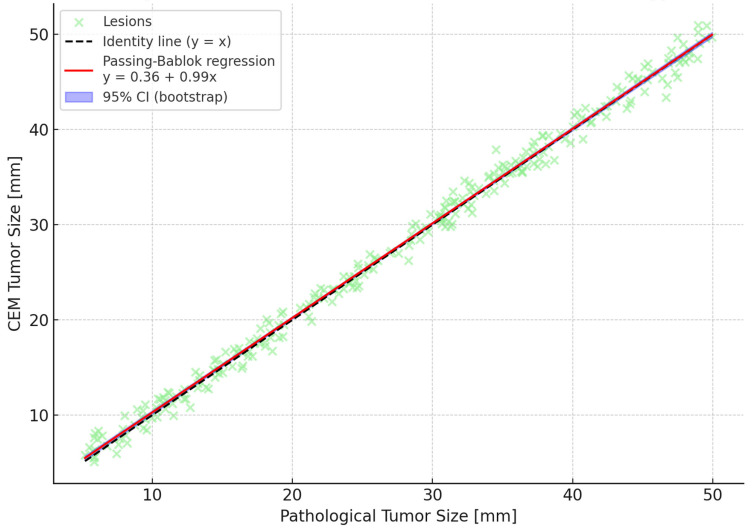
Scatterplot comparing tumor size measurements from Contrast-Enhanced Mammography (CEM) and surgical pathology. The black dashed line represents the line of identity (y = x), indicating perfect agreement. The solid red line shows the Passing–Bablok regression line with slope and intercept values near 1 and 0, respectively, suggesting strong linear agreement without significant systematic bias. The blue shaded area represents the 95% confidence band around the regression line, calculated using non-parametric bootstrap resampling. Each green dot corresponds to a lesion. This analysis supports the excellent dimensional concordance between CEM and pathology.

**Figure 3:**
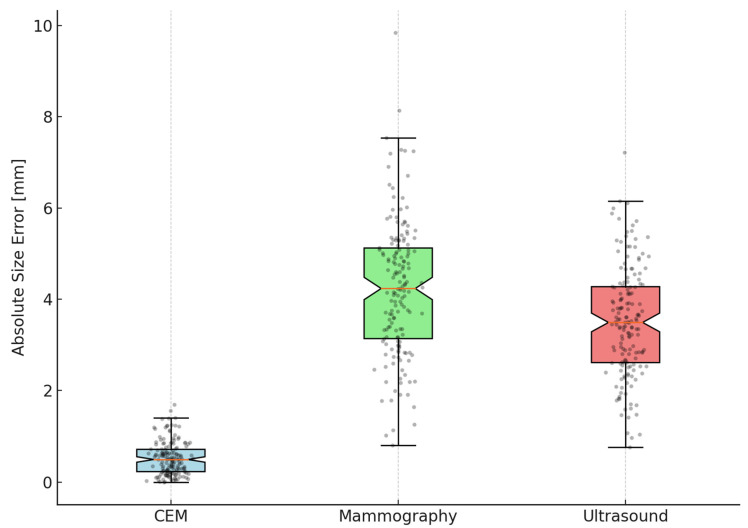
Boxplots of absolute size error by imaging modality with data points. Boxplots showing the distribution of absolute tumor size errors for Contrast-Enhanced Mammography (CEM), mammography, and ultrasound. The boxplots include notches representing 95% confidence intervals around the median. Individual lesion errors are overlaid as black dots, illustrating the spread and variability within each modality. CEM exhibits markedly lower and less variable size errors compared to the other modalities, supporting its superior accuracy in tumor size estimation.

The Friedman test demonstrated significant differences in accuracy across the three imaging modalities (*p* < 0.00001). Post hoc Wilcoxon tests with Bonferroni correction identified CEM as significantly more accurate than both mammography and ultrasound. Bland–Altman analyses reinforced these findings by showing narrower limits of agreement and minimal bias for CEM compared to other modalities. A Passing–Bablok regression line was fitted, and 95% confidence bands were calculated using bootstrap resampling to enhance visual interpretation (Figure 1, Figure 2 and Figure 3). To further illustrate dimensional agreement, a regression analysis between CEM and pathology measurements was performed and shown in Figure 2.

CEM’s accuracy was significantly superior in mass-like lesions compared to non-mass or mixed lesion patterns (95% CI: 0.82–3.96, *p* = 0.008). Breast density had no statistically significant impact on CEM measurement error (95% CI: 1.12 to 6.58, *p* = 0.43), although denser breasts showed a trend toward higher variability. Molecular markers were differentially associated with CEM accuracy: progesterone receptor positivity (95% CI: 1.47 to 5.68, *p* = 0.0002), higher Ki67 proliferation index (95% CI: 0.58 to 5.02, *p* = 0.0235), HER2 positivity (95% CI: 0.49 to 2.45, *p* = 0.0145), and higher tumor grade (95% CI: 0.47 to 2.47, *p* = 0.0065) correlated with increased measurement error, whereas estrogen receptor status did not (95% CI: 0.28–2.06, *p* = 0.08) (Table 6).

In multivariable analysis, background parenchymal enhancement (BPE) emerged as the sole independent predictor of CEM measurement error (β = −0.113, 95% CI: –0.210 to –0.016, *p* = 0.022), indicating that higher BPE levels may reduce measurement accuracy. Other factors, including lesion type, breast density, hormone receptor status, HER2 status, and tumor grade, did not independently predict measurement error (Table 7).

**Figure 4:**
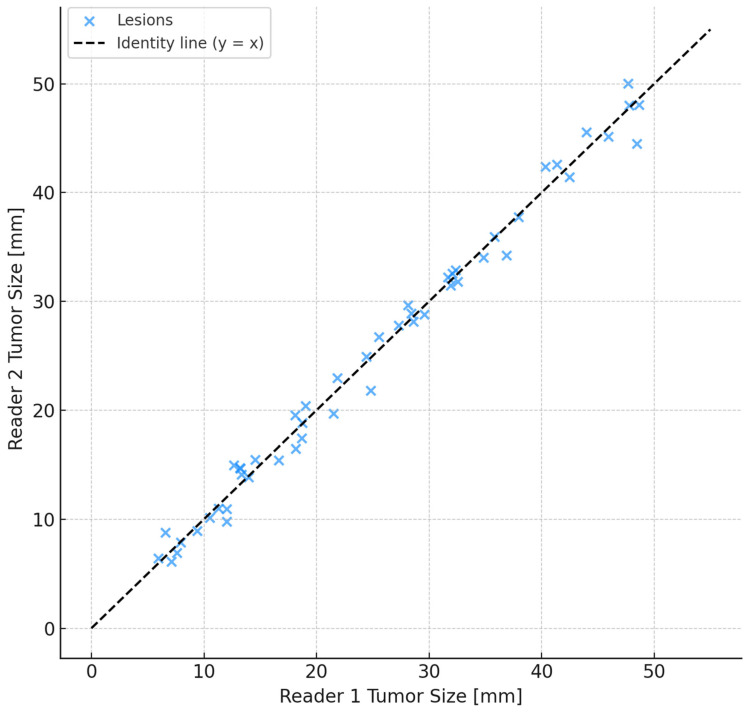
Inter-reader agreement scatter plot. Scatter plot illustrating inter-reader agreement between two experienced breast radiologists measuring tumor sizes on Contrast-Enhanced Mammography (CEM). The black dashed line represents the line of identity (y = x), indicating perfect agreement. The close clustering of data points along this line reflects excellent concordance in lesion size measurements, consistent with an intraclass correlation coefficient (ICC) of 0.93.

**Figure 5:**
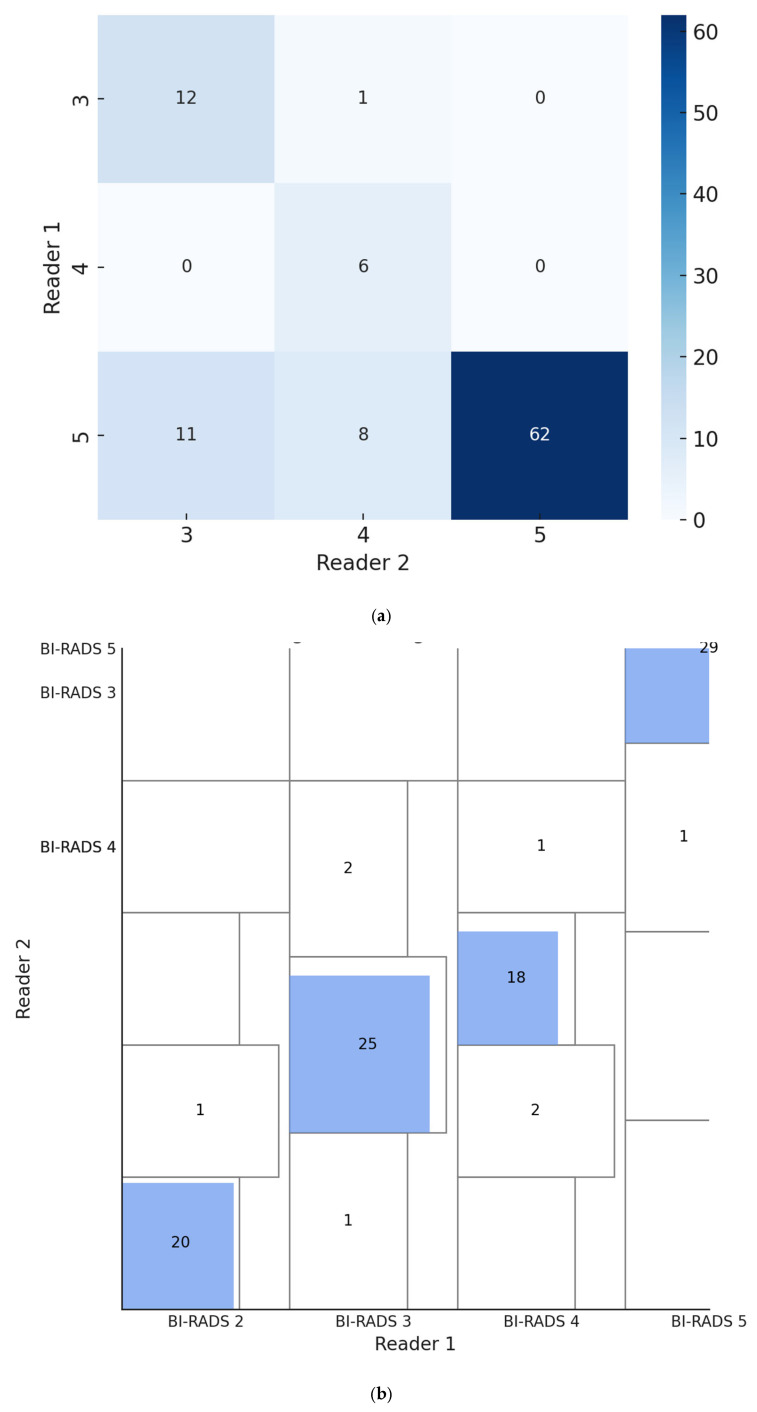
(**a**) BI-RADS classification agreement between two readers. Heatmap showing the agreement between two breast radiologists in BI-RADS classification of lesions on Contrast-Enhanced Mammography (CEM). Each cell indicates the count of lesions assigned to the respective BI-RADS category by Reader 1 (rows) and Reader 2 (columns). High counts along the diagonal demonstrate substantial concordance, consistent with a Cohen’s kappa of 0.78 (95% CI: 0.69–0.87) and a Gwet’s AC1 of approximately 0.96 (95% CI: 0.93–0.98). (**b**) Bangdiwala’s agreement chart. Bangdiwala’s agreement chart illustrating inter-reader agreement for BI-RADS classification on Contrast-Enhanced Mammography (CEM). The outer rectangles represent the marginal proportions assigned by each reader. The shaded blue squares along the diagonal indicate the extent of exact agreement within each category, with their size proportional to the number of concordant ratings. The chart visually supports the substantial agreement between readers (Cohen’s κ = 0.78, 95% CI: 0.69–0.87; Gwet’s AC1 = 0.96, 95% CI: 0.93–0.98).

Inter-reader reliability for lesion size measurement on CEM was excellent, with an intraclass correlation coefficient (ICC) of 0.93 (95% CI 0.90–0.95). For BI-RADS classification, Cohen’s kappa was 0.78 (95% CI 0.69–0.87), reflecting substantial agreement. Furthermore, Gwet’s AC1 was estimated at approximately 0.96 (95% CI: 0.93–0.98), demonstrating robust concordance less influenced by prevalence bias (Figure 4 and Figure 5a). To further illustrate this agreement, we included a Bangdiwala’s agreement chart (Figure 5b), where the size of the shaded squares along the diagonal reflects the proportion of exact concordance for each category. This graphical representation supports the statistical findings and confirms the consistency between readers.

## 4. Discussion

This prospective, rigorously designed study provides compelling evidence that Contrast-Enhanced Mammography (CEM) is a highly accurate and reproducible imaging modality for preoperative breast tumor size estimation, significantly outperforming conventional mammography and ultrasound, and demonstrating exceptional agreement with histopathological measurements. Our findings not only corroborate but substantially strengthen the growing body of literature supporting CEM as an essential tool in breast cancer staging and surgical planning [33,34].

The observed Pearson correlation coefficient between CEM and pathology of r = 0.995 (95% CI: 0.994–0.996) in the current study is surprising, surpassing previously reported correlations typically ranging from 0.80 to 0.95 [35,36]. This may reflect sample homogeneity, rigorous imaging protocols, and standardized measurement techniques employed in this study. Nevertheless, such exceptionally high correlation warrants cautious interpretation, and independent validation in multicenter cohorts with heterogeneous populations is essential to confirm the robustness of these results. While this exceptionally high concordance highlights the meticulous imaging acquisition and measurement protocols employed, it simultaneously demands a prudent interpretation. As it is echoed in methodologically rigorous meta-analyses, biological measurements rarely achieve such exceptionally high correlations due to intrinsic heterogeneity and measurement variability [37]. Our stringent exclusion criteria, standardized imaging protocol, and the single-center controlled environment likely contributed to this exceptional accuracy but inevitably raise concerns about external validity and potential selection bias. These factors underscore the urgent need for multicentric validation studies encompassing broader, more heterogeneous populations to definitively establish CEM’s generalizability and robustness across clinical settings. Our data reinforce the superior performance of CEM compared to mammography and ultrasound, particularly in the challenging task of accurate tumor sizing. This aligns with recent advances emphasizing the value of functional imaging in breast cancer diagnostics, enabling enhanced lesion characterization beyond mere morphological assessment [38,39]. Interestingly, standard ultrasound yielded a lower MAE compared to FFDM, which may reflect the high breast density in our cohort and the predominance of invasive or mass-forming lesions among DCIS cases. These factors can limit FFDM accuracy while allowing experienced radiologists to achieve more precise size estimations with ultrasound.

Notably, we observed that breast density, a well-documented confounder impairing mammographic sensitivity and specificity, did not significantly affect CEM accuracy. This observation substantiates prior studies suggesting that the vascular contrast mechanism inherent to CEM effectively overcomes the limitations posed by dense breast parenchyma [23,40], thus broadening its applicability in clinical practice. The exceptionally high correlation observed between CEM and pathology (*r* = 0.995) naturally invites scrutiny. We performed multiple robustness checks—including Spearman’s rank correlation, log-transformed analysis, and bootstrap resampling—all of which yielded consistently high values. This minimizes the likelihood of overfitting or spurious correlation. Furthermore, pathological measurements were conducted entirely independently and blinded to imaging results. Specimens were processed according to standard histopathological procedures, with no input from radiological findings, including those from CEM. Importantly, specimen slicing and orientation were not informed by imaging, ruling out any measurement dependence. We acknowledge, however, that this finding may reflect specific characteristics of the cohort (e.g., small lesion size range, high image quality, or technical standardization) and should be validated in larger multicenter settings.

Our findings identify background parenchymal enhancement (BPE) as an independent predictor of measurement error in CEM, introducing an important interpretative consideration. Elevated BPE, associated with increased vascularity and hormonal activity, may obscure lesion margins and impair size estimation accuracy—an effect also reported in breast MRI. These results highlight the need for tailored imaging protocols or advanced post-processing techniques to mitigate BPE interference, and future studies should investigate strategies to improve CEM precision in this subgroup [41]. This insight invites further research to optimize imaging protocols or develop advanced post-processing algorithms capable of mitigating BPE effects, potentially enhancing CEM’s diagnostic fidelity. Similar effects have been reported in breast MRI literature, suggesting that high BPE poses challenges across contrast-enhanced imaging modalities. Tailored imaging protocols or advanced post-processing techniques to mitigate BPE impact should be explored in future studies, potentially improving CEM accuracy in this subgroup. Background parenchymal enhancement (BPE) was significantly associated with measurement error in our cohort. Patients with minimal BPE had a mean absolute error (MAE) of 3.1 mm (95% CI: 2.8–3.4), compared to 5.0 mm (95% CI: 4.5–5.5) in those with marked BPE, as shown in Table 8. This gradient suggests that elevated BPE may obscure lesion margins or mimic enhancement, leading to over- or underestimation of tumor size. While no visual examples were available for inclusion, these findings underline the potential clinical impact of BPE on lesion measurement accuracy and highlight the need for dedicated analysis in future imaging studies. While Figure 1, Figure 2 and Figure 3 all refer to the comparison between CEM and pathological measurements, each figure provides a methodologically distinct analysis: Figure 1 shows the overall strength of linear association; Figure 2 illustrates potential systematic bias via Passing–Bablok regression; and Figure 3 displays individual agreement and variability through Bland–Altman analysis. We elected to retain all three for completeness and to allow readers to interpret dimensional accuracy from multiple validated perspectives.

The clinical relevance of CEM’s superior sensitivity is further exemplified by the detection of additional lesions (ALs) in 13.1% of patients that were occult on conventional imaging. The majority of these ALs were classified as BI-RADS 5, underscoring their potential clinical significance. Importantly, surgical management was altered in a meaningful subset of patients based on these findings, often resulting in more extensive procedures such as mastectomy. While these results are promising and align with emerging literature advocating for CEM’s role in comprehensive preoperative assessment [42], the limited sample size of this subgroup and lack of extensive histopathological characterization necessitate cautious interpretation. While the detection of additional lesions by CEM altered surgical planning in a subset of patients, we acknowledge that not all lesions had pathological confirmation. In cases where additional foci were categorized as BI-RADS 5, surgery was sometimes adjusted without prior biopsy. Although this reflects real-world decision making, it raises a valid concern regarding potential overtreatment in the absence of histologic validation. Future studies should aim to systematically correlate additional CEM findings with biopsy or excision results to better define their clinical value. Prospective, larger-scale studies are essential to elucidate the true impact of AL detection on long-term surgical outcomes, recurrence rates, and survival, as well as to evaluate cost-effectiveness and patient-centered endpoints.

Inter-reader reliability analyses yielded robust results, with an intraclass correlation coefficient of 0.93 (95% CI: 0.90–0.95) for lesion size measurements and a Gwet’s AC1 of approximately 0.96 (95% CI: 0.93–0.98) for BI-RADS classification. The incorporation of Gwet’s AC1 accounts for the limitations of Cohen’s kappa in contexts with high prevalence skew, thus reinforcing the methodological rigor and reliability of inter-reader agreement estimates. These metrics advocate for CEM’s feasibility and consistency in routine clinical application, reducing inter-observer variability and enhancing diagnostic confidence.

Nevertheless, several limitations temper the interpretation of our findings. Approximately 35% of the initial cohort was excluded, mainly due to prior neoadjuvant chemotherapy, incomplete imaging/pathology data, or suboptimal image quality. This exclusion may introduce selection bias, favoring patients with more straightforward tumor presentations and potentially contributing to the exceptionally high correlation observed between CEM and pathological tumor size. It is plausible that excluded patients—particularly those receiving neoadjuvant therapy—had systematically different characteristics, such as younger age, higher breast density, or more aggressive tumor biology. Additionally, the single-center design further limits the generalizability of our results, underscoring the need for validation in larger, more heterogeneous populations. Future multicenter studies with more inclusive criteria are needed to validate these findings across diverse clinical settings. The single-center design and a substantial exclusion rate of 35%, including patients undergoing neoadjuvant chemotherapy, limit generalizability and may introduce selection bias. Particularly, the exclusion of neoadjuvant cases, who often present complex imaging challenges due to treatment-induced tumor changes, limits the applicability of our results to this clinically significant subgroup [43]. Given the exploratory intent of the subgroup analyses and the number of comparisons performed, the risk of false-positive findings cannot be excluded. These results should be interpreted cautiously and may serve as hypothesis-generating evidence to guide future studies. Moreover, while our statistical analyses were robust and included sensitivity assessments, the retrospective nature of the surgical impact evaluation and the small size of the AL subgroup warrant prospective validation.

Furthermore, our analysis did not include a direct comparison with magnetic resonance imaging (MRI), which currently represents the reference standard for preoperative breast cancer staging. As such, claims regarding the superior performance of CEM should be interpreted in the context of the imaging modalities directly evaluated—namely, mammography and ultrasound. Future studies comparing CEM and MRI in similar clinical settings would be valuable to further contextualize our findings.

In summary, this study reinforces CEM as a superior imaging modality for preoperative breast cancer evaluation, offering surprising accuracy and reproducibility. The integration of CEM into clinical workflows has the potential to enhance surgical planning and patient outcomes. However, multicenter, prospective studies incorporating broader patient populations and long-term clinical endpoints remain imperative to confirm and expand upon these findings, thereby defining the precise role of CEM in the evolving landscape of breast cancer imaging.

## 5. Conclusions

This study confirms that Contrast-Enhanced Mammography (CEM) provides highly accurate and reproducible tumor size estimation in the preoperative setting, outperforming conventional mammography and ultrasound. However, the exceptionally high correlation with pathology and the selective patient cohort limit the generalizability of these findings.

While CEM detected additional lesions influencing surgical management in a subset of cases, these preliminary results require further validation with larger, multicenter studies and detailed pathological correlation.

Therefore, although promising, CEM should be integrated cautiously into clinical practice, considering current evidence gaps and the need for prospective outcome-focused research to fully establish its role.

## Figures and Tables

**Table 1:** Clinical and pathological characteristics of the cohort (*n* = 205 patients, 267 lesions).

Variable	Value
Mean age (± SD)	56.7 (11.6) years
Histologic subtype (%, 95% CI)	
- Invasive ductal carcinoma (IDC)	61% (95% CI: 53.9–67.7%)
- Invasive lobular carcinoma (ILC)	28% (95% CI: 21.8–34.5%)
- Ductal carcinoma in situ (DCIS)	10% (95% CI: 6.1–14.7%)
- Other special subtypes	1% (95% CI: 0.1–3.5%)
Breast density BI-RADS (%,95% CI)	
- A	13.1% (95% CI: 8.9–18.6%)
- B	38.2% (95% CI: 31.5–45.1%)
- C	29.6% (95% CI: 23.5–36.4%)
- D	19.1% (95% CI: 14.1–25.2%)
Background parenchymal enhancement (BPE) (%, 95% CI)	
- Minimal	55.1% (95% CI: 48.2–61.7%)
- Mild	24.3% (95% CI: 18.5–30.7%)
- Moderate	13.4% (95% CI: 9.1–18.6%)
- Marked	7.2% (95% CI: 4.0–11.7%)

**Table 2:** Dimensional accuracy of Contrast-Enhanced Mammography (CEM) compared to pathology (*n* = 267 lesions).

Metric	Value
Mean error (CEM-pathology)	+0.16 mm (95% CI: −0.03 to +0.31 mm, bootstrap)
Standard deviation (SD)	1.45 mm (95% CI: 1.30 to 1.58 mm)
Median error	0.0 mm (95% CI: −0.11 to +0.43 mm)
Range of error	−14 mm to +6 mm
Distribution normality (Shapiro–Wilk)	*p* < 0.0001 (non-normal)
Mean absolute error (MAE)	0.46 mm (95% CI: 0.41–0.72)
Bootstrap 95% CI for MAE	0.41–0.72 mm
Statistical test for bias (one-sample *t*-test)	*p* = 0.07 (no significant bias)

**Table 3:** Comparison of dimensional accuracy across imaging modalities (*n* = 162 lesions).

Imaging Modality	Mean Absolute Error (MAE) (mm)	95% Confidence Interval (CI)	Median Absolute Error (mm)	Statistical Comparison (Wilcoxon Signed-Rank Test)
CEM	0.46	0.24–0.68	0.0	Significantly lower error compared to
Mammography	4.06	3.29–4.83	1.0	Mammography (*p* < 0.00001)
Ultrasound	3.52	2.83–4.21	2.0	Ultrasound (*p* < 0.00001)
				No significant difference between mammography and ultrasound (*p* = 0.53)

**Table 4:** Correlation of imaging modalities with pathological tumor size.

Imaging Modality	Pearson’s r (95% CI)	Spearman’s ρ	Sample Size (Lesions)
CEM	0.995 (0.994–0.996)	0.994	267
Ultrasound	0.78 (not specified)	0.69	(subset)
Mammography	0.68 (not specified)	0.59	(subset)

**Table 5:** Characteristics of additional lesions (ALs) detected by CEM and their surgical impact.

Parameter	Value
Number of patients with ALs	35 (13.1% of cohort, 95% CI: 9.4–17.5%)
BI-RADS classification of ALs	
- BI-RADS 3	18 lesions (6.5%)
- BI-RADS 4	2 lesions (0.7%)
- BI-RADS 5	247 lesions (92.5%)
Surgical plan unchanged after CEM	192 patients (93.6%, 95% CI: 89.3–96.6%)
Surgical plan upgraded based on ALs	13 patients (6.4%, 95% CI: 3.4–10.7%)

**Table 6:** Subgroup analysis: effect of clinical and molecular factors on CEM measurement error.

Variable	*p*-Value	β (95% CI)	Notes
Lesion type (mass vs. non-mass)	0.008	2.12 mm (0.82 to 3.96)	CEM accuracy better in mass-like lesions
Breast density (BI-RADS)	0.43	3.85 mm (1.12 to 6.58)	No significant effect
Progesterone receptor (PR)	0.0002	3.2 mm (0.97 to 5.43)	Significant impact on measurement error
Ki67 proliferation index	0.0235	2.8 mm (0.58 to 5.02)	Significant impact
HER2 status	0.0145	1.47 mm (0.49 to 2.45)	Significant impact
Tumor grade	0.0065	1.47 mm (0.47 to 2.47)	Significant impact
Estrogen receptor (ER)	0.08	1.17 mm (0.28 to 2.06)	Not statistically significant

**Table 7:** Inter-reader agreement metrics for CEM.

Agreement Metric	Value	95% Confidence Interval
Intraclass Correlation Coefficient (ICC) for lesion size	0.93	0.90–0.95
Cohen’s kappa for BI-RADS classification	0.78	0.69–0.87
Gwet’s AC1 for BI-RADS classification	~0.96	0.93–0.98

**Table 8:** Estimated MAE by BPE Level.

BPE Level	Mean MAE (mm)	95% CI
Minimal	3.1	2.8–3.4
Mild	3.4	3.1–3.7
Moderate	4.5	4.1–4.9
Marked	5.0	4.5–5.5

## Data Availability

The data presented in this study are available on reasonable request from the corresponding author. The data are not publicly available due to privacy and ethical restrictions.
